# The Strategies of Pathogen-Oriented Therapy on Circumventing Antimicrobial Resistance

**DOI:** 10.34133/2020/2016201

**Published:** 2020-09-28

**Authors:** Zifang Shang, Siew Yin Chan, Qing Song, Peng Li, Wei Huang

**Affiliations:** ^1^Frontiers Science Center for Flexible Electronics (FSCFE), Xi'an Institute of Flexible Electronics (IFE) & Xi'an Institute of Biomedical Materials and Engineering (IBME), Northwestern Polytechnical University (NPU), Xi'an 710072, China; ^2^Key Laboratory for Organic Electronics and Information Displays (KLOEID) and Institute of Advanced Materials (IAM), Nanjing University of Posts and Telecommunications (NUPT), Nanjing 210023, China; ^3^Key Laboratory of Flexible Electronics (KLOFE) & Institute of Advanced Materials (IAM), Jiangsu National Synergetic Innovation Center for Advanced Materials (SICAM), Nanjing Tech University (NanjingTech), Nanjing 211816, China

## Abstract

The emerging antimicrobial resistance (AMR) poses serious threats to the global public health. Conventional antibiotics have been eclipsed in combating with drug-resistant bacteria. Moreover, the developing and deploying of novel antimicrobial drugs have trudged, as few new antibiotics are being developed over time and even fewer of them can hit the market. Alternative therapeutic strategies to resolve the AMR crisis are urgently required. Pathogen-oriented therapy (POT) springs up as a promising approach in circumventing antibiotic resistance. The tactic underling POT is applying antibacterial compounds or materials directly to infected regions to treat specific bacteria species or strains with goals of improving the drug efficacy and reducing nontargeting and the development of drug resistance. This review exemplifies recent trends in the development of POTs for circumventing AMR, including the adoption of antibiotic-antibiotic conjugates, antimicrobial peptides, therapeutic monoclonal antibodies, nanotechnologies, CRISPR-Cas systems, and microbiota modulations. Employing these alternative approaches alone or in combination shows promising advantages for addressing the growing clinical embarrassment of antibiotics in fighting drug-resistant bacteria.

## 1. Introduction

After several decades of successful practices using antibiotics to treat bacterial infectious diseases, the emergence of antimicrobial resistance (AMR) has been recognized as a global public health crisis nowadays [1–4]. At present, antibiotic-resistant bacteria kill 700,000 people/year worldwide, and the annual death toll caused by AMR is expected to be 10 million by 2050, disbursing about $100 trillion globally [5, 6]. When microbes develop multidrug- or extensively drug resistance (MDR or XDR), they are known as “superbugs” [7]. In facing the rise of antibiotic resistance, the World Health Organization (WHO) released its first priority list of bacteria in urgent need of new antibiotics in early 2017. The list includes 12 dangerous bacterial families that threaten human health, with an objective to guide and promote the research and development of new antibiotics [8]. However, the growth rate of bacterial drug resistance tends to be underestimated and is much faster than the development rate of new antibiotics [9]. This is mainly due to the overuse and misuse of antibiotics to treat infections. Moreover, the development of new antibiotics is slow due to unsatisfactory clinical data, such as unexpected pharmacokinetic parameters, poor stability, low permeability, and lack of *in vivo* activity and efficiency [10, 11]. Though extensive research is ongoing, very limited new antibiotics can make their way to the patients [12].

Thus, alternative therapeutic approaches to resolve this issue of AMR have attracted increasing research interests in recent years. The principle behind these approaches is to circumvent bacterial resistance against antibiotics by applying antimicrobial compounds or materials directly to specific bacterial species, strains, or infected sites. We believe these strategies can be generally categorized as pathogen-oriented therapy (POT). POT shows a promise in targeting the specific bacteria, increasing effective drug concentration, and reducing the dosage of antibiotics, thus improving the antibacterial efficacy over traditional antibiotics, while reducing nontargeting effect and slowing down the development of drug resistance. These POT strategies include the conjugation among antibiotics, exploitation of antimicrobial peptides (AMPs), adoption of bacteria-specific antibodies, utilization of nanotechnologies, employment of CRISPR-Cas systems, and involvement of microbiota modulation. In this review, we described the research progresses of these POT strategies, elucidating their characteristics and challenges associated with their applications in the future.

## 2. Antibiotic-Antibiotic Conjugates (AACs)

With the emergence of drug-resistant bacteria, advancing the development of antibiotics is more critical than ever [13]. Creating new antibiotics or developing alternative therapeutic approaches are important to prevent serious drug-resistant bacterial infections [14]. Analysis shows that there are minute amount of new antibiotics targeting most of the world's dangerous infections [15]. Historical data shows that the success rate of clinical drug development is low that only one-fifth of the products will be approved for phase I clinical trials [16]. To date, about 44 new antibiotics are under clinical development. Of these drugs, only 12 have the potential to address the three key carbapenem-resistant Gram-negative pathogens (viz. *Enterobacteriaceae*, *Pseudomonas aeruginosa*, and *Acinetobacter baumannii*) on the WHO's priority list of antibiotic-resistant Gram-negative pathogens [8, 17]. Researchers have tried hard to develop advanced substitutes by coupling existing antibiotics to overcome drug resistance, known as the antibiotic-antibiotic conjugates (AACs) [18–23]. These AACs block the action mode of antibiotic resistance or enhance the overall inhibitory effect of antibiotics [24, 25]. Depending on the functional properties of coupling groups, AACs can be classified into quinolone/fluoroquinolone, aminoglycoside, *β*-lactamase inhibitor, and macrolide conjugates.  Table 1 lists some examples of antibacterial applications of AACs.

### 2.1. Quinolone/Fluoroquinolone Conjugates

Quinolones/fluoroquinolones are broad-spectrum antibiotics against both Gram-negative and Gram-positive bacteria [45–47]. Fluoroquinolones are effective in some life-threatening bacterial infections such as *Legionella pneumophila* infection. The antibacterial activity of fluoroquinolones is achieved by inhibiting the catalytic cycle of the bacterial topoisomerase, which controls the topological state of the deoxyribonucleic acid (DNA). Bacterial topoisomerase is an indispensable component of basic cellular processes such as DNA replication and transcription, representing a critical targeting site for therapeutic purpose [47].

Oxazolidinones are a class of synthetic antibiotics that inhibit the initiation of protein biosynthesis by binding to the V region of the 23S rRNA catalytic center of the ribosomal 50S subunit in the peptidyl transferase [48, 49]. Oxazolidinones have been demonstrated to be a higher antibacterial activity against Gram-positive bacteria compared with Gram-negative bacteria, and oxazolidinones conjugated with fluoroquinolones using a chemical coupling method increased its antibacterial activity and spectrum [50]. MCB3681 is a quinolone-oxazolidinone conjugate (QOC) developed by Morphochem [26] and exhibited antibacterial activity with minimum inhibitory concentration (MIC) values ranging from 0.06 to 1 *μ*g/mL against several strains of Gram-positive pathogens such as methicillin-resistant *Staphylococcus aureus* (MRSA), methicillin-sensitive *S. aureus* (MSSA), and vancomycin-resistant *enterococci* (VRE) [51, 52]. A recent study also reported that MCB3681 displayed good antibacterial activities against *Clostridium difficile in vitro* with no evidence of drug resistance in the isolated strains [27]. Cadazolid represents a more advanced QOC with an oxazolidinone pharmacophore replaced by a fluoroquinolone moiety [28]. It is initially developed by Actelion Pharmaceuticals to treat *C. difficile* infection [28, 53], exhibiting antibacterial activity with the MIC range of 0.125 to 0.5 *μ*g/mL [30]. Cadazolid inhibits protein synthesis by oxazolidinone domain and restrains RNA synthesis by quinolone moiety [29, 54]. Another influential quinolone conjugate is quinolone-rifampicin conjugate, CBR-2092, developed by Cumbre Pharmaceuticals through linking 4H-4-oxo-quinolizine and rifamycin SV pharmacophore via a hydrazide group [31, 32]. CBR-2092 showed fairly good antimicrobial properties against clinically isolated Gram-positive bacteria such as MRSA, and its activity was better than quinolone (ciprofloxacin) alone [31]. However, its antibacterial activity against Gram-negative bacteria such as *Escherichia coli* was considered mild and not comparable to that of ciprofloxacin. Functional studies demonstrated that CBR-2092 exhibited an inhibitory effect on ribonucleic acid (RNA) polymerase, DNA gyrase, and DNA topoisomerase IV [32].

### 2.2. Aminoglycoside Conjugates

Aside from quinolone/fluoroquinolone conjugates, aminoglycosides are also widely used as conjugated antibiotics [55–57]. Aminoglycosides are broad-spectrum antibiotics that are active against most aerobic and facultative anaerobic Gram-negative bacteria [58, 59] by inhibiting protein synthesis by binding to 16S rRNA on ribosomal 30S subunit [60, 61]. However, aminoglycosides are highly hydrophilic [62] and tend to exhibit poor cell permeability. Their pharmacokinetic parameters can be improved by coupling with other types of antibiotics. In particular, aminoglycoside neomycin B was linked with ciprofloxacin via 1,2,3 benzotriazole ligands [33]. The conjugated molecule demonstrated improved antibacterial activity against Gram-negative bacteria, Gram-positive MRSA, and even the most prevalent resistant types related to aminoglycosides, compared with pristine neomycin B. The inhibition mechanism of neomycin B-ciprofloxacin on bacterial protein synthesis is similar to that of neomycin B, while the conjugates exhibited 32 times stronger inhibitory activity on DNA gyrase and topoisomerase IV compared with paternal ciprofloxacin [33]. Besides, tobramycin and moxifloxacin conjugates were used to treat *Pseudomonas aeruginosa* infections [34]. The coupling of tobramycin and moxifloxacin enhanced the permeability of antibiotics to the outer membrane of pathogenic bacteria. The conjugate protected *Galleria mellonella larvae* from the lethal effects of MDR *P. aeruginosa*. Drug resistance selection studies showed that the use of tobramycin-moxifloxacin conjugate induced lower probability of drug resistance of *P. aeruginosa* compared to their parental antibiotics [34]. Aminoglycoside-modifying enzymes catalyze phosphorylation, acetylation, or adenylation of hydroxyl and amino groups of aminoglycosides, resulting in the inactivation of aminoglycosides. The coupling of antibiotics with aminoglycoside could inhibit the enzymic modification of aminoglycosides [35, 63]. Hanessian et al. simulated the conjugate of neomycin and sisomicin by cheminformatics, and the MIC values of the conjugate against several aminoglycoside-resistant *P. aeruginosa*, *E. coli*, *A. baumannii*, and *S. aureus* were reduced by nearly 64 times compared with the parental neomycin B or sisomicin [36].

### 2.3. *β*-Lactamase Inhibitor Conjugates

The *β*-lactamase inhibitors are also a kind of antibiotic with extensive influence. There are mainly three kinds of *β*-lactamase antibiotic conjugates: avibactam involved conjugates (e.g., ceftazidime-avibactam, aztreonam-avibactam, and ceftaroline fosamil-avibactam), vaborbactam involved conjugates (e.g., meropenem-vaborbactam), and relebactam involved conjugates (e.g., imipenem-relebactam) [21, 64–68].

Among the avibactam conjugates, ceftazidime-avibactam has been used in Europe and the United States for the treatment of adults with complicated urinary tract infections (e.g., pyelonephritis and hospital-acquired pneumonia). It is also approved recently by the European Drug Administration for adult patients infected with aerobic Gram-negative bacteria with limited treatment regimen [38, 69]. Meropenem-vaborbactam is the first carbapenem/*β*-lactamase inhibitor conjugate recently approved by the US Food and Drug Administration (FDA) for the treatment of complicated urinary tract infections and acute pyelonephritis [39, 40]. Vaborbactam, also known as RPX7009, is a nontoxic cyclic boric acid *β*-lactamase inhibitor but lacks antibacterial activity *in vitro* [70]. The boric acid group covalently bounds to the serine side chain at the *β*-lactamase catalytic site [71], inhibiting the activity of *β*-lactamase. Vaborbactam has anti-Ambler class A and C enzyme activities [72]. It has been shown to inhibit various class A carbapenem enzymes (KPC-2, KPC-3, KPC-4, BKC-1, FRI-1, and SME-2), class A extended-spectrum *β*-lactamases (ESBL) (CTX-M, SHV, and TEM), and class C cephalosporinase (CMY, P99). However, it has no inhibitory activity against D-type carbapenem (OXA-48) or metallo-*β*-lactamases (NDM, VIM, and IMP) [65, 73]. Relebactam-imipenem is currently being evaluated for the treatment of Gram-negative bacterial infections, including hospital-acquired bacterial pneumonia, ventilator-associated bacterial pneumonia, complex intraperitoneal infection, and urinary tract infection caused by MDR pathogens, especially *P. aeruginosa* and carbapenemase-producing *E. coli*, *K. pneumoniae*, and *Enterobacter* [74–76]. Prescription of imipenem combined with relebactam for the treatment of Gram-negative bacterial infections is currently in phase III of a clinical trial [41].

### 2.4. Macrolide Conjugates

Macrolide is a general term for natural products of polyketides and their semisynthetic derivatives [77]. They are composed of macrocyclic lactone of different ring sizes and are attached with one or more deoxy sugars or amino sugars. Macrolides play an important role in inhibiting bacteria via reaction with bacterial 50S ribosomal subunits and interfering with the protein synthesis pathway [78]. Due to their high-affinity binding ability with bacterial ribosomes and the highly conserved structure of ribosomes in almost all bacterial species, macrolides are endowed with broad-spectrum antibacterial properties as antibiotics [79]. Since the discovery of macrolide erythromycin in 1950, many derivatives have been synthesized, including the famous azithromycin and clarithromycin [80–83]. The emergence of many macrolide-resistant bacterial strains and the long-term laborious development process of conventional new drugs have rendered the development in creating new conjugates based on existing drugs. Through this means, the coupled molecules could synergistically possess unique biological characteristics and play a better role in antibacterial clinical use.

Azithromycin-sulfonamide showed good antibacterial activity *in vitro* with MIC values ranging from 0.5 to 2 *μ*g/mL against macrolide-resistant *Streptococcus pyogenes* and *Streptococcus pneumoniae* strains, but inactive against Gram-negative *H. influenzae* and *E. coli* strains [43]. The macrolide-quinolone conjugates have improved antibacterial activity against drug-resistant strains, and further modification enhanced their effectiveness [44]. These molecules have made a breakthrough in antibacterial activity, showing better antibacterial activity than telithromycin, successfully inhibiting various macrolide-resistant bacterial isolates. The optimized conjugate showed fairly good antibacterial activities against MDR *S. pneumoniae* and *S. pyogenes* with a resistance phenotype of less than 0.125 *μ*g/mL MIC.

In summary, AAC can be composed of two antibiotics with both of their antimicrobial activity for exerting a synergistic effect, or be composed of antibiotics with their bioactive adjuvants, such as inhibitors of efflux pumps or antibiotic-modifying enzymes, for enhancing the effect of antibiotics and improving their pharmacokinetic parameters. Despite these advantages, AAC pays more attention to whether it has superior or at least no inferior antibacterial activity compared with classical combination therapy or parental generation, but most current studies are lack of systematic research on the occurrence and mechanism of drug resistance.

## 3. Antimicrobial Peptides (AMPs) in Combatting AMR

### 3.1. AMPs

AMPs are oligomers of amino acids that have homogeneous structural groups, which are differed from the small molecule of antibiotics described in the previous section. Due to AMPs' significant antibacterial role in host organisms, a large number of clinical trials have demonstrated AMPs as promising candidates for AMR [84, 85]. Depending on the structural characteristics of AMPs, they are classified into four types: *α*-helix, *β*-sheet, extended, and cyclic. Most natural AMPs are modified in order to improve their metabolic stability, bioavailability, safety, immunogenicity, etc. [86–88]. Most AMPs are cationic and amphiphilic, allowing them to penetrate and/or destroy bacterial membranes. Identifying the optimum ratios of cationic and hydrophobic amino acid residues is important for maintaining low hemolysis and high antibacterial activity [89, 90]. AMPs target on the bacterial membrane or intracellular components to achieve antibacterial effect [91, 92]. Interestingly, AMPs do not interact with specific targets in pathogens [93], which render the evolution of pathogen resistance to AMPs at a relatively slow rate. Few cases of drug resistance to AMPs have been reported, particularly tyrothricin, which has been clinically used for more than 60 years [94]. Since 1939, a natural antimicrobial peptide named gramicidin has been identified from *Bacillus brevis* that exhibited antibacterial activity against Gram-positive bacteria in the infected wound sites in guinea pigs as a substitute for synthetic antibiotics [95]. Since then, researchers have developed various types of AMPs to combat the prickly problem of antibiotic resistance, e.g., *α*-helix (magainin [96], LL-37 [97, 98], and hCAP18 [99]), *β*-sheet (defensin [100], protegrin [101], and tachyplesin [102]), and extended (indolicidin [103] and bactenecin [104]).

**Figure 1:**
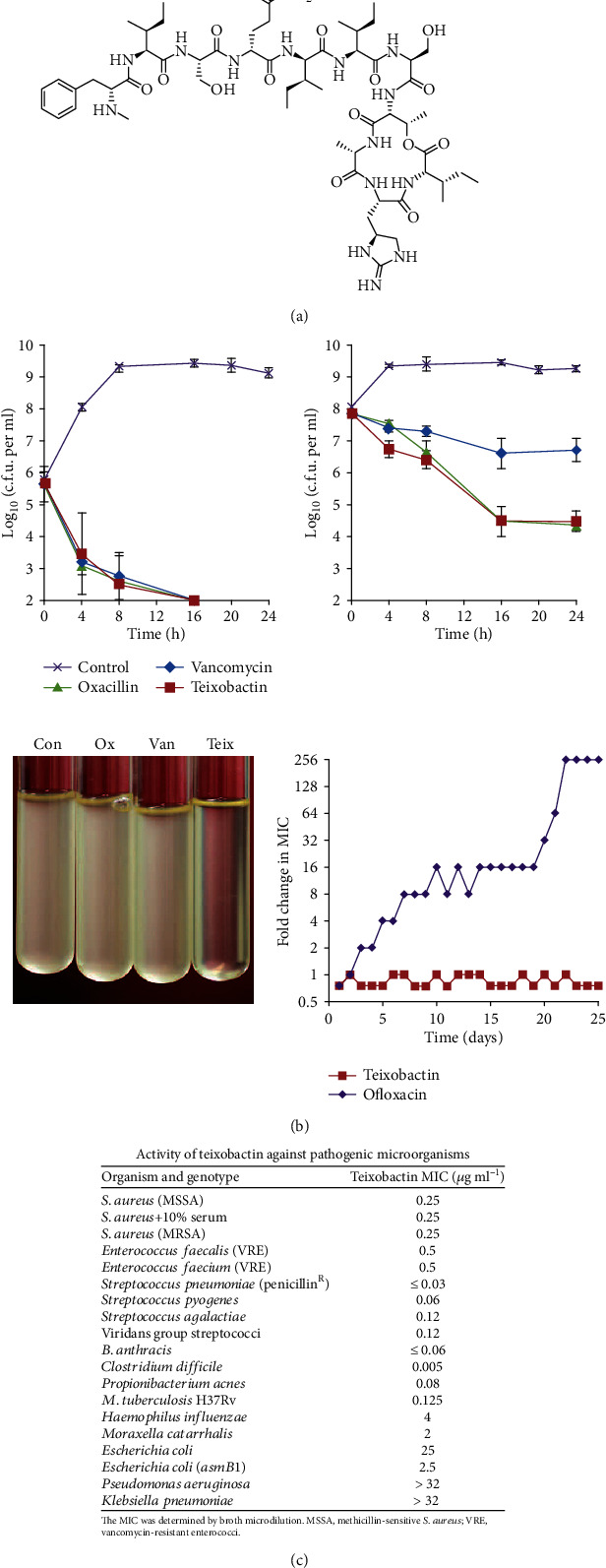
The structure of teixobactin and its performance against pathogenic microorganisms. (a) Schematic structure of teixobactin. (b) Activity of teixobactin against pathogenic microorganisms. (c) Time-dependent killing of pathogens by teixobactin. *S. aureus* grew to early (upper left) and late (upper right) exponential phase and challenged with antibiotics. Teixobactin treatment resulted in lysis (lower left) and resistance acquisition during serial passaging in the presence of sub-MIC levels of antimicrobials (bottom lower right). The *y*-axis is the highest concentration the cells grew during passaging [126]. *Copyright © 2020, Springer Nature.*

Natural AMPs are easily degraded by proteolytic enzymes or peptidases or cleared by the liver and kidney in the body, hindering their development in clinical treatment against pathogens [105, 106]. To improve the metabolic stability and bioavailability of natural AMPs, modification and synthesis of AMPs have been adopted. Modification approaches can be classified into three forms: cyclization [107–109], replacement of noncanonical residues [110–112], and N/C-terminal modifications [88, 113–115]. Most oral active peptides are cyclic, including the well-known potent peptide antibiotics such as bacitracin A [116, 117], colistin [118, 119], gramicidin [120], and polymyxins B1 and B2 [121, 122], demonstrating that cyclization of AMPs is effective [123]. Cardoso et al. have pioneered the use of D-amino acids in place of L-amino acids to improve the bioavailability of AMPs. The modified AMPs had higher proteolytic stability while having similar antibacterial activity as the original AMPs [124]. Isomerization has since become a popular method to increase the stability of AMPs against proteolysis. N/C-terminal acetylation, amidation, or addition of hydrophobic oligomers can also be employed to increase AMPs' resistance to peptidase or protease hydrolysis *in vivo*. Lewis et al. successfully extracted teixobactin from the Gram-negative bacterium *Eleftheria terrae* with the new iChip [125] technology [126]. Teixobactin is a compound that potently inhibits the growth of *S. aureus* (Figure 1). It is a polypeptide consisting of 11 amino acids, comprising nonprotein amino acid enduracididine, methylphenylalanine, and four D-amino acids (Figure 1(a)).

Teixobactin displayed a fairly good inhibitory effect on Gram-positive bacteria (including the difficult-to-treat *Enterococcus* and *Mycobacterium tuberculosis*) with MIC value of <1 *μ*g/mL for most bacteria (Figure 1(b)). It particularly inhibited the growth of *C. difficile* and *Bacillus anthracis* with MIC values of 5 and 20 ng/mL, respectively. It showed higher bactericidal activity against *S. aureus* compared to vancomycin in killing late exponential phase populations. Teixobactin also displayed bactericidal activity against vancomycin-intermediate *S. aureus* (VISA) (Figure 1(c)). However, teixobactin had no activity against most Gram-negative bacteria. It only had an inhibitory effect on a mutant *E. coli* that lacks the outer membrane permeation barrier. Surprisingly, no drug-resistant strain, hemolysis, and genotoxicity *in vivo* were detected for the use of teixobactin [126]. To some extent, teixobactin has excellent antibacterial activity after modification via cyclization, replacement of noncanonical residues, and N-terminal modifications of amino acids, showing promising roles in POT in the future.

**Figure 2:**
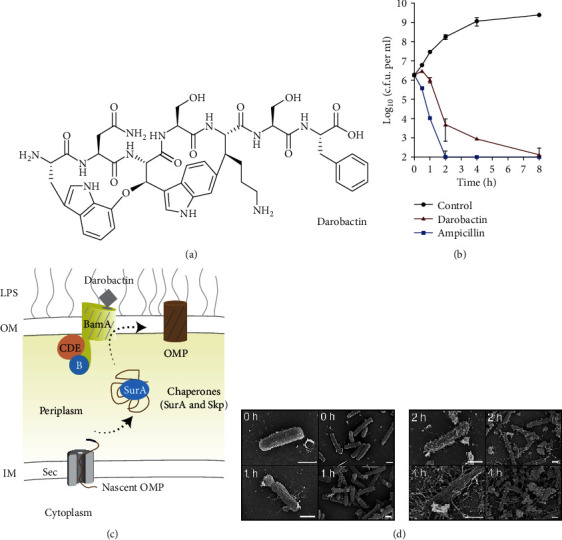
Darobactin produced by a silent operon of *P. khanii* is a bactericidal antibiotic. (a) Darobactin structure. (b) Time-dependent killing of *E. coli* MG1655 by darobactin. An exponential culture of *E. coli* MG1655 was challenged with 16x MIC antibiotics. (c) Schematic of the Bam complex. IM: inner membrane; OM: outer membrane. (d) Scanning electron microscopy analysis of *E. coli* MG1655 treated with 16x MIC darobactin. Scale bars, 1 *μ*m [127]. *Copyright © 2020, Springer Nature.*

**Figure 3:**
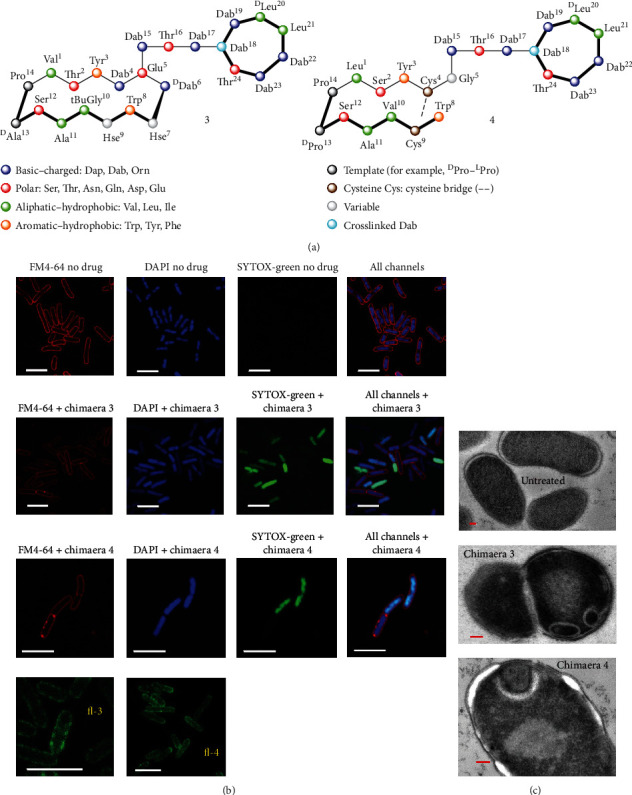
The structures of the chimeric polymyxin B1 and their mechanism studies using fluorescence and transmission electron microscopy. (a) The structures of chimeric polymyxin B1. (b) Fluorescence microscopy of *E. coli* ATCC 25922 cells grown in MH-II and stained with FM4-64, DAPI, or SYTOX-green, without treatment. (c) Transmission electron microscopy of *E. coli* ATCC 25922 untreated or grown with 3 or 4 at concentrations causing about 50% growth inhibition (about 0.1 mg L^−1^) (*n* = 3 biologically independent experiments). Scale bars, 200 nm [128]. *Copyright © 2020, Springer Nature.*

Darobactin is a modified heptapeptide with an amino acid sequence of W1-N2-W3-S4-K5-S6-F7W1-N2-W3-S4-K5-S6-F7. It is a potential new antibiotic screened from *Photorhabdus* isolates (Figure 2) [127]. The amino acids within darobactin are linked through two macrocycles. Darobactin demonstrated a fairly good effect on Gram-negative pathogens (*E. coli*, *K. pneumoniae*, *Pseudomonas aeruginosa*, *Acinetobacter baumannii*, etc.) without cytotoxicity *in vitro*and *in vivo* (Figures 2(a) and 2(b)). Mechanism study revealed that darobactin binds to the BamA protein located in the outer membrane of Gram-negative bacteria (Figure 2(c)), destructing the outer membrane structure and inducing the death of bacteria (Figure 2(d)). On the other hand, Luther et al. synthesized a series of antibiotics inspired by the scaffold of natural products. These chimeric antibiotics contain *β*-hairpin macrocycles linked to the macrocycles found in the natural products polymyxin and colistin families (Figure 3(a)) [128]. The optimized derivatives have bactericidal activity against a wide range of Gram-negative ESKAPE pathogens, including MDR and EDR *E. coli* strains (Figures 3(b) and 3(c)). It also exhibited low cytotoxicity to mammalian cells, having a low possibility in inducing drug resistance in all tested bacterial strains. The synthesized antibiotics also maintained good levels of potency in the presence of human serum, showing decent safety and pharmacokinetic characteristics. Mechanism study demonstrated that they bind to the outer membrane protein BamA, leading the abnormal growth of bacteria and eventually causing the death of bacteria. These derivatives showed strong *in vivo* efficacy in mouse models of peritonitis induced by colistin-resistant *E. coli* strains containing *mcr-1* and *mcr-3* drug-resistant genes and in mouse models of thigh infected with drug-resistant *E. coli*, *A. baumannii*, and *P. aeruginosa*.  Table 2 lists some selected AMPs' development at different clinical status.

### 3.2. AMP-Antibiotic Conjugates

Most microbial surface contains heaps of negatively charged compounds such as lipoteichoic acid and lipopolysaccharide [129–131], while mammal cell surfaces are rich in zwitterionic phospholipids, cholesterol, and sphingomyelin compounds [132, 133]. This major difference allows AMPs to selectively target microbial cells, providing the foundation for the design and development of AMP conjugate drugs. From the perspective on antibacterial mechanism, both compounds of conjugated AMPs would have contributed antibacterial activities, possibly enhancing the overall effect. The AMP moieties could possibly increase the accumulation of the second conjugated group through different carrier mechanisms, ideally enhancing the antibacterial activity against Gram-positive and Gram-negative bacteria [134, 135]. As for AMP-antibiotic conjugates, AMPs could act as uptake enhancers and/or antimicrobial agent, which is a promising strategy for POT.

Magainin II is a typical representative of *α*-helical AMP, which is isolated from *Xenopus laevis*. It mainly binds to the surface of the anionic lipid layer through electrostatic interaction [136], resulting in membrane thinning and destruction and inducing pore formation and lysis of cells [137, 138]. The conjugate magainin II-vancomycin demonstrated higher activity against vancomycin-resistant *Enterococci* compared to vancomycin alone [139]. Other peptides such as M33, indolicidine, or transactivating transcriptional activator (TAT) have been conjugated with levofloxacin for POT [140]. M33 is a branched synthetic peptide with a broad-spectrum antibacterial effect by binding lipopolysaccharide (LPS) on the surface of Gram-negative bacteria [141]. Experimental results suggested that the activity of M33-levofloxacin conjugate was not superior to that of M33, but the combination of M33 with levofloxacin was better than that of M33 and levofloxacin alone. Indolicidine is an AMP-rich cationic tryptophan in bovine neutrophils and is active against both Gram-positive and Gram-negative bacteria [142, 143]. Transactivating transcriptional activator (TAT) is a positively charged dodecapeptide derived from human immunodeficiency virus 1 (HIV-1). TAT is rich in arginine and lysine, enhancing cell membrane permeability [144]. Indolicidine-levofloxacin and TAT-levofloxacin conjugates have also been reported to have similar antibacterial properties [145].

To improve the selective antibacterial effect, oligonucleotides and antibiotics can be conjugated. Triclosan is a sulfuryl-CoA reductase inhibitor and has been reported that it is not able to inhibit *Toxoplasma gondii* [146]. By incorporating oligoarginines, the conjugate oligoarginines-triclosan demonstrated an inhibitory effect against the growth of *Toxoplasma gondii in vitro* and *in vivo*. Besides, Schmidt et al. coupled the penetrating peptide Pen with tobramycin [147]. The conjugates not only retained high antibacterial activity of tobramycin but also had improved efficiency in killing *S. aureus* and *E. coli*. When 25 *μ*M of tobramycin was used in conjugation with peptide Pen, the efficiency of the conjugates in killing *S. aureus* and *E. coli* was increased by 106 times and 104 times, respectively. Excitingly, there were no side effects induced on eukaryotes. Hansen et al. investigated the coupling of antisense peptide nucleic acid (PNA) with AMP. PNA is a new class of antibiotics that inhibit the growth of bacteria by specifically knocking out the expression of essential genes [148]. Studies have shown that some AMPs such as buforin 2-A (BF2-A), drosocin, Pep-1-K, and KLW-9 with intracellular antimicrobial functions can be used as effective carriers for bacteria to deliver antibacterial PNA and target the *acpP* gene essential for fatty acid synthesis. Pep-1-K and KLW-L-9 have been identified as peptide moieties that can display normal activity without relying on the bacterial endomembrane transporter SbmA at MIC concentration of 2-4 mM [148].

In summary, using AMPs alone as antimicrobial agents or AMPs as uptake enhancers is a promising strategy, which can not only kill extracellular pathogens but also target intracellular pathogens. In fact, the AMPs component of the AMP-antibiotic conjugates may increase the antibacterial activity of the drug against intracellular microorganisms and reduce the cytotoxicity to mammalian cells. Despite the above advantages, some of the disadvantages of AMP conjugates, including high production cost, low oral bioavailability, poor metabolic stability, and undesirable serum stability and immunogenicity, still need to be further studied before they can be used *in vivo*. In general, this drug delivery strategy is expected to provide new treatment options for the fight against pathogen resistance.

## 4. Antibody Therapy in Combatting AMR

### 4.1. Monoclonal Antibody (mAb) Therapy

The therapeutic potential of serum therapy, which essentially uses antibodies in the plasma of rehabilitated patients to neutralize bacteria, has been recognized since late 19^th^ century when Shibasaburo Kitasato and Emil von Behring used serum from patients infected with *Corynebacterium diphtheria* and *Clostridium tetanus* to prevent infection in other patients or horses [149]. In the early 20^th^ century, people began to use serum therapy to treat viral and bacterial diseases [150]. With the emergence of antibiotics in the 1930s, serum therapy suffered an eclipse [151]. As antibiotics are easier to manufacture and the cost of production is cheaper, they have led the development in fighting bacterial infections over the past 80 years [152]. However, the widespread emergence of antibiotic-resistant bacteria poses a major threat to the public health. Moreover, most bacterial infections are caused by antibiotic-resistant pathogens. The development of antibody therapy is one of the promising alternatives for antibiotics to have a direct clinical impact due to the unique advantages of mAbs in recognizing specific pathogens [153–156]. mAb usually target antigens exposed on the surface of the pathogen or toxins secreted by the pathogen, which are not targeted by antibiotics. Unlike broad-spectrum antibiotics, which have no selectivity on bacterial composition, mAb is highly specific and exert ecological protection effect on nontargeted bacteria. mAb also helps in reducing the usage of conventional antibiotics, diminishing the selection pressure for resistant strains.

There are several mechanisms of action for the antibacterial effect of mAb. The first proposed mechanism involves mAb bind to the target antigen on the surface of the pathogen, changes the conformation of the fragment crystallizable (Fc) region [157], and promotes complement component 1q (C1q) to recognize and bind to the antibody-binding site. Subsequently, the complement activation system is activated to form a membrane attack complex [158], which eventually leads to bacterial cell lysis and death [158–160]. mAb 17H12 and 8F12 have been isolated from mice inoculated with a mixture of anthrax protective antigen coupled with *K. pneumoniae* capsular polysaccharide (CPS). These two mAbs specifically bind to the CPS epitope of the carbapenem-resistant *K. pneumoniae* strain ST258 [161], promoting the phagocytosis and cellular cytotoxicity of human neutrophils and mouse macrophages against *K. pneumoniae*. Chimeric mAb 2C7 with a E430G Fc (2C7-E430G Fc) also demonstrated a similar mechanism of killing bacteria [162]. Besides, mAb can also promote the killing of the opsonophagocytic through the binding of the Fc region with the receptor on the surface of phagocytes [163, 164]. MRSA is a common cause of deadly blood infections. It produces coagulase (Coa) to activate the host of prothrombin fibrinogen [165]. The R domain in the C terminal of Coa comprises 27 amino acid residues with conservative tandem repeat sequences, which binds fibrinogen onto the surface of MRSA to protect pathogens from phagocytosis by immune cells [166, 167]. Thomer et al. immunized mice with full-length Coa expressed by *S. aureus* as an antigen and obtained mAb 3B3 [168]. It was demonstrated that the R domain of Coa bound onto 3B3, inhibiting Coa binding to fibrinogen and triggering phagocytosis of *Staphylococcus*. Nielsen et al. extracted mAb C8 by inoculation of mice with a sublethal dose of highly toxic *A. baumannii* strain [169]. C8 targeted and bound to the carbohydrate of bacterial surface capsule to enhance opsonophagocytosis. After humanized, C8 significantly increases the phagocytosis of *A. baumannii*. Finally, mAb neutralizes the activity of bacterial toxins by inhibiting the binding of bacterial exotoxin molecules with host cell targets or blocking their polymerization with other toxin subunits [170–172]. Many pathogenic bacteria cause diseases by releasing toxins, and the use of therapeutic antibodies that neutralize these toxins is an integral part of the treatment for infections. Anthrax toxin is a three-component protein exotoxin composed of a protective antigen and two enzymes (edema factor and lethal factor) segments [173], working together to suppress the immune response and kill the host cells. Obiltoxaximab (Elusys Therapeutics) and Raxibacumab (AstraZeneca) are obtained via the phage display technique that can neutralize the protective antigen of *B. anthracis*, inhibiting the lethal effect of anthrax toxin [174, 175]. Interleukin A/B (LukAB) is a recently discovered toxin in *S. aureus* infections, which kills human primary phagocytes and is the main factor for human monocyte death [176]. Three monoclonal antibodies (SA-13, SA-15, and SA-17) were obtained from B cells of a 12-year-old patient with *S. aureus* osteomyelitis via the hybridoma technique [177] and were reported to possess a unique neutralization mechanism against the virulence factor LukABv of *S. aureus* and demonstrated competent efficacy *in vivo*.  Table 3 shows antibodies against pathogenic bacteria in clinical research.

### 4.2. Antibody-Antibiotic Conjugates

The design of antibody-antibiotic conjugates is similar to the design of antibody-drug conjugates (ADCs) [197–200] for the treatment of tumor cells. Using antibodies specific for bacterial cell surface antigens, these conjugates were endowed with specificity and efficacy that cannot be afforded by traditional drugs.

**Figure 4:**
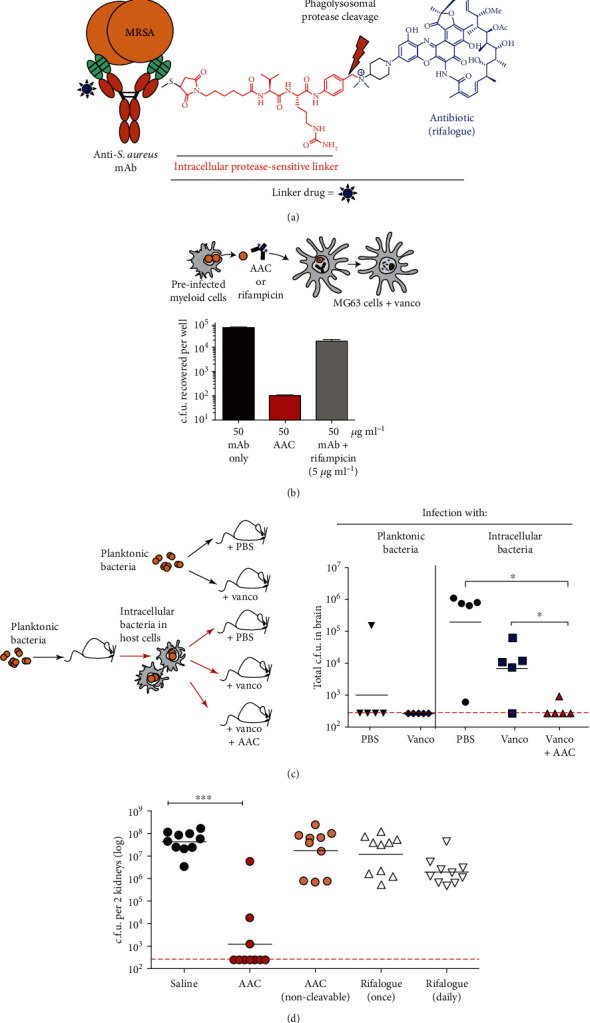
The model of novel of antibody-antibiotic and its antibacterial properties. (a) Model of AAC. (b) Intracellular USA300 were added to a monolayer of MG63 with antibody, AAC, or a mixture of antibody plus rifampicin in media containing vancomycin (vanco). Surviving bacteria were enumerated 24 h later. (c) AAC kills intracellular reservoirs of MRSA *in vivo*. (d) 10 mice per group were injected with human IgG to achieve a concentration of 10 mg/mL, then infected with MRSA. Treatment as indicated was begun 24 h after infection [155]. *Copyright © 2015, Springer Nature.*

Genentech has reported a striking strategy on treating intracellular persistent *S. aureus* infections recently (Figure 4) [155]. A group of specific anti-*S. aureus* antibodies had been screened against several MRSA strains derived from 40 patients. The specific antibodies bind selectively to glycopolymers on the outer layer of Gram-positive bacteria known as wall-teichoic acids (WTAs). The verified antibodies were coupled with rifamycin derivative (rifalogue) to kill MRSA hidden in cells. Rifalogue was attached to the specific antibody of WTA with a linker, enabling it to be cleaved by lysosomal cathepsin and subsequently the releasing of it (Figure 4(a)). Unlike the mixture of rifampicin and unconjugated anti-MRSA antibody, the antibody-antibiotic conjugate significantly reduced the transfer of *S. aureus* from infected peritoneal cells to uninfected osteoblasts in the presence of vancomycin (Figure 4(b)). Mice injected with vancomycin and intracellular methicillin-resistant *S. aureus* formed bacterial colonies in the brain but were effectively eliminated in mice treated with vancomycin and the conjugate (Figure 4(c)). In addition, a single dose of the conjugate also helped in preventing kidney colonization, whereas unconjugated rifalogue, unconjugated WTA-specific antibody, or noncleavable conjugate treatments in the control group did not (Figure 4(d)).

In summary, mAb therapy is a promising way to reduce drug resistance and economic burden of clinical infectious bacteria in the current situation of widespread antibiotic resistance and rarely developed new antibiotics for the treatment of drug-resistant bacteria. Although the development of mAb is increasing over time, only a few mAbs have been evaluated in clinical studies; more research into mAb is needed to expand the potential of it in combating AMR. The strategy using antibody-antibiotic conjugates for treating infectious diseases is an exciting approach by combining the pharmacological properties of antibodies and antibiotics into a single molecule. Given that some of the effective antibiotics in clinical trials fail *in vivo* due to poor pharmacokinetics or undesirable host toxicity, antibody-antibiotic conjugates can often help overcome these problems by targeted delivery of antimicrobial compounds to infected cells. Therefore, although this combination is technically challenging and expensive, its potential for specialized treatment of intracellular pathogens is very promising.

## 5. Nanotechnology in Combatting AMR

Advances in nanotechnology open up promising antimicrobial nanotherapies, particularly in the development of nanoparticles in drug delivery, have had a major impact in combating AMR [201–206]. Nanoparticles (NPs) are tiny particles less than 100 nm in at least one dimension, and their typical small size allows them to be absorbed by phagocytes and introduce antimicrobial agents into the mammalian cells, targeting intracellular pathogens [207]. NPs have high specific surface areas and functional structures, enabling the highest possible loading capacity of drug molecules [208, 209]. In addition, the high adaptability of NPs to drug molecules (i.e., hydrophobic and hydrophilic) and their high stability in physiological fluids permit controlled biodegradation and minimize adverse side effects [210]. These desirable characteristics allow improvement in bioavailability and therapeutic effect of antibacterial drugs, making NPs as promising drug carriers. Due to their inherent antimicrobial properties and their admirable physicochemical (delivery or maintenance of antimicrobial agents at specific sites of infection) properties, NPs have attracted much attention in POT research.

### 5.1. Antimicrobial NPs

NPs such as silver, copper, and gold show good antibacterial activity and have a wide range of applications [211–213]. However, these inorganic NPs do not exhibit antibacterial activity with selectivity, rendering the development of composite NPs. Graphene oxide-silver (GO-Ag) NPs differentially inhibited Gram-negative *E. coli* and Gram-positive *S. aureus* [214]. GO-Ag NPs exhibited a bacteriostatic effect for *E. coli* and *S. aureus* by destroying the integrity of the bacterial cell wall and inhibiting the cell division cycle, respectively. Aminosaccharide-based gold NPs were developed to work according to the difference in cell wall structure between Gram-positive and Gram-negative bacteria [215]. This NP composite demonstrated narrow-spectrum antibacterial activity, restricting the growth of Gram-positive bacteria by specifically inhibiting the biosynthesis of Gram-positive bacteria cell wall. It can minimize the damage on probiotics and prevent dysbacteriosis. Aminosaccharide-based gold NPs had also shown great potential for wound healing application [215].

At normal physiological conditions, pathogen cell walls are generally negatively charged [216], and the electrostatic interactions of the bacterial cell wall can be targeted by designing positively charged NPs. A cationic polymer such as chitosan can be incorporated into NPs' systems in order to improve the efficiency of antibacterial activity. In a study involving grafting of cationic polymer chitosan and small molecule 2-mercapto-1-methylimidazole onto the surface of gold nanoparticles, the resulting NPs bestowed multivalent interaction with a bacterial membrane with improved antibacterial activity [217]. The cationic polymer coatings targeted selected bacteria and exhibited antibacterial activity by destroying the bacterial cell plasma membrane, inhibiting bacterial proliferation, and preventing biofilm formation via strong electrostatic interactions with the negatively charged bacterial membrane. The composite NPs were capable of adhering to the surface of mature biofilms and inactivated the surrounded bacterial cells, causing the biofilm to rupture [216]. Most importantly, chitosan-based gold NPs displayed great biocompatibility by exhibiting selective antimicrobial activity on bacteria but remaining harmless to mammalian cells.

### 5.2. Antibiotic-Delivering NPs

NPs play a promising role in the targeted delivery of antibiotics, providing a platform for POT to fight against the dilemma of antibiotic resistance. Precise drug delivery can be achieved through stimuli-responsive NPs. As a result of the combined activities of bacterial metabolism and host immune response, acidification can occur at the infected sites when hosts were infected by bacteria [218, 219]. The efficacy of the delivery and release of drugs can be significantly hindered by the acid environment. To tackle the problem, surface charge-switching polymeric NPs were developed using poly(d, l-lactic-*co*-glycolic acid)-b-poly(l-histidine)-b-poly(ethylene glycol) (PLGA-PLH-PEG) [220]. The NPs were at first kept at pH 7.4 (negative charge) to prevent it from interacting with nontargeted objects. Under low pH condition, the imidazole group in the PLH can be partially protonated and bind tightly with bacteria for drug delivery. Delivery of drugs can be terminated by alleviating the pH value of the environment. This represents alternatives for targeted therapy with acidic Gram-positive, Gram-negative, or plague infections [220].

There are other unique infectious microenvironments that can be used as a targeted means of antibiotics at the sites of bacterial infections. Recent research has looked into antibiotic treatment strategy involving macrophages for bacterial infections [221]. Mannose ligands were grafted onto the polyphosphoester core made of polyethylene glycol (PEG) to form nanogels, and these nanogels can be used as drug carrier-targeted macrophages. It was reported that macrophages express a high level of mannose receptors [222], allowing drug accumulation at the bacterial infection sites and subsequent degradation of the NPs by bacterial generated active phosphatase or phospholipase. The nanogels with antibiotics were reported to effectively inhibit the growth of MRSA. The survival rate of zebrafish infected with MRSA showed that mannosylated nanogel-encapsulated vancomycin was better than the treatment with nonmannose-treated nanogels. Mannosylated nanogels have successfully attained both the ability to target macrophages and lesion site-activatable drug release properties, enhancing the inhibition of bacterial growth [221].

**Figure 5:**
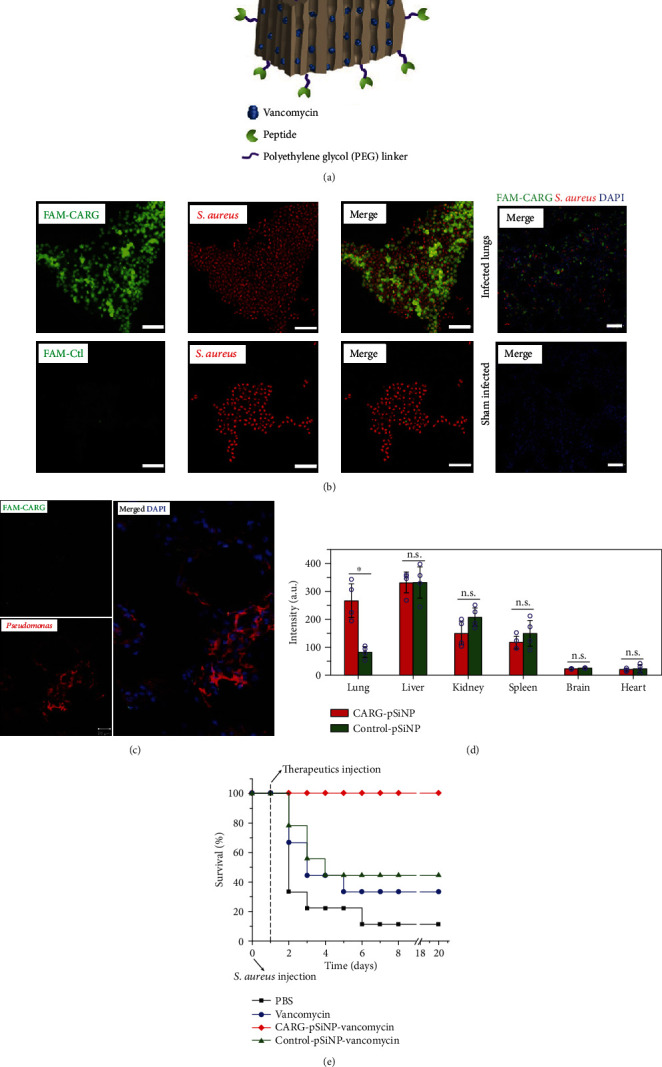
The schematic structure and antibacterial efficacy of targeted antibiotic-loaded nanoparticles. (a) A schematic illustration of the therapeutic nanoparticle system consisting of pSiNP, chemotherapeutic agent, and homing peptide. (b) The CARG peptide shows selective binding to cultured *S. aureus in vitro* and homes to infected lungs *in vivo*. (c) The CARG does not recognize cultured *Pseudomonas aeruginosa* bacteria or lung tissue infected with these bacteria. (d) Biodistribution of nanoparticles obtained from the luminescence intensity in each organ. (e) Survival rate (*n* = 9) of mice after intratracheal inoculation with 5 × 10^7^ colony-forming units (CFU) of *S. aureus* [224]. *Copyright © 2018, Springer Nature.*

Precise drug release through NPs with targeted molecules is also an effective antimicrobial treatment. Ghanbar et al. achieved selective bactericidal efficacy against MRSA by encapsulating biocide (C17) in solid lipid nanoparticles (SLNPs) and coupling MRSA-specific antibody (Ab) to the surface of the SLNPs [223]. The antibacterial activity of SLNPs loaded with Ab (C17-SLNP-Ab) was better than SLNPs loaded without Ab (C17-SLNP) and C17-SLNP with nonspecific IgG (C17-SLNP-IgG). C17-SLN-Ab showed selective toxicity to MRSA in the coculture assay of MRSA/fibroblast. The toxicity of C17-SLN-Ab to MRSA was higher than that of *P. aeruginosa*. The authors have also successfully adjusted the selectivity from MRSA to *E. coli* by changing C17 to K12, showing the versatility of this new strategy [223]. Hussain et al. obtained a *S. aureus*-specific 9-amino-acid oligopeptide by phage display technology (Figure 5) [224]. The obtained CARGGLKSC(CARG) was attached to NPs loaded with vancomycin (Figure 5(a)), enabling nanoparticles to specifically target *S. aureus*-infected tissues, achieving precise drug delivery, reducing the systemic dose needed, minimizing side effects, and enhancing antimicrobial activity. CARG specifically bound to *S. aureus* and selectively accumulated in the lungs and skin of mice infected with *S. aureus* (Figure 5(b)) rather than in noninfected tissues and tissues infected with *Pseudomonas* bacteria (Figures 5(c) and 5(d)). *In vivo* experiments have shown that the NPs with specific oligopeptide are more effective in inhibiting *S. aureus* infection than equivalent doses of nontargeted vancomycin NPs or without vancomycin (Figure 5(e)) [224]. This strategy can reduce the dosage of antibiotics needed and attenuate side effects and the risk of drug resistance.

Furthermore, Zhang et al. developed bioresponsive NPs for targeted delivery of drugs which achieved effective control and treatment of sepsis [225]. A pH/enzyme-sensitive amphiphilic polymer was synthesized and self-assembled to form nanomicelles. These nanomicelles effectively loaded antibiotics ciprofloxacin (CIP) and anti-inflammatory drugs ((2-[(aminocarbonyl)amino]-5-(4-fluorophenyl)-3-thiophenecarboxamide). The surface of the drug-loaded NPs was further modified by incorporating intercellular adhesion molecule-1 (ICAM-1), a targeting antibody via specific action of biotin-avidin. Multiple animal models were studied to elucidate the controlled release mechanism of drug delivery at infectious microenvironments (IMEs). In a mouse model of sepsis infected by *P. aeruginosa*, the developed NPs effectively eliminated invading bacteria and alleviated inflammation, thereby increasing the survival rate of mice. This study provides new insights on the mechanism of NPs in the treatment of infectious diseases and presents new ideas for developing new functional nanomaterials based on disease characteristics.

In brief, with rapid development in nanotechnology and a more in-depth study of infectious diseases that have been conducted in recent years, antibacterial drugs have made significant progress in targeted delivery. Most of the current research focuses on the fundamentals of nanoparticle-based pathogen-oriented therapy, mainly targeting to improve the therapeutic effectiveness and reduce drug resistance. At present, antimicrobial NPs are still rarely used in clinical settings; yet, they have great potentials in the future treatment of various infectious diseases.

## 6. CRISPR-Cas System in Combatting AMR

### 6.1. CRISPER-Cas

The CRISPR system is an acquired immune defense mechanism that evolved from the constant attack of foreign viruses or plasmids [226]. It was originally discovered in bacteria and archaea. To date, more than 40 different Cas protein families have been reported, each of which differs significantly in the synthesis of crRNA, the integration of spacer sequences, and the way in which foreign DNA is cleaved [227]. The CRISPR/Cas system can be classified into two classes and six types: class one which has a more complex structure (types I, III, and IV) and class two that has a simpler structure multiple (types II, V, and VI). Class one Cas proteins are involved in the process of exogenous DNA recognition and cleavage, while class two is recognized and cleaved by a single multidomain enzyme [228]. In eukaryotic cells, cleaved DNA is efficiently repaired by ubiquitous mechanisms such as homologous recombination or nonhomologous end joining [229]. In contrast, bacteria cannot perform nonhomologous end-joining mechanism and cannot repair DNA double-stranded cleavage by CRISPR/Cas nuclease, triggering their death. Using the CRISPR/Cas system to precisely cut the DNA of bacteria can lead to the development of a new, efficient, and specific method for eliminating bacteria [230].

**Figure 6:**
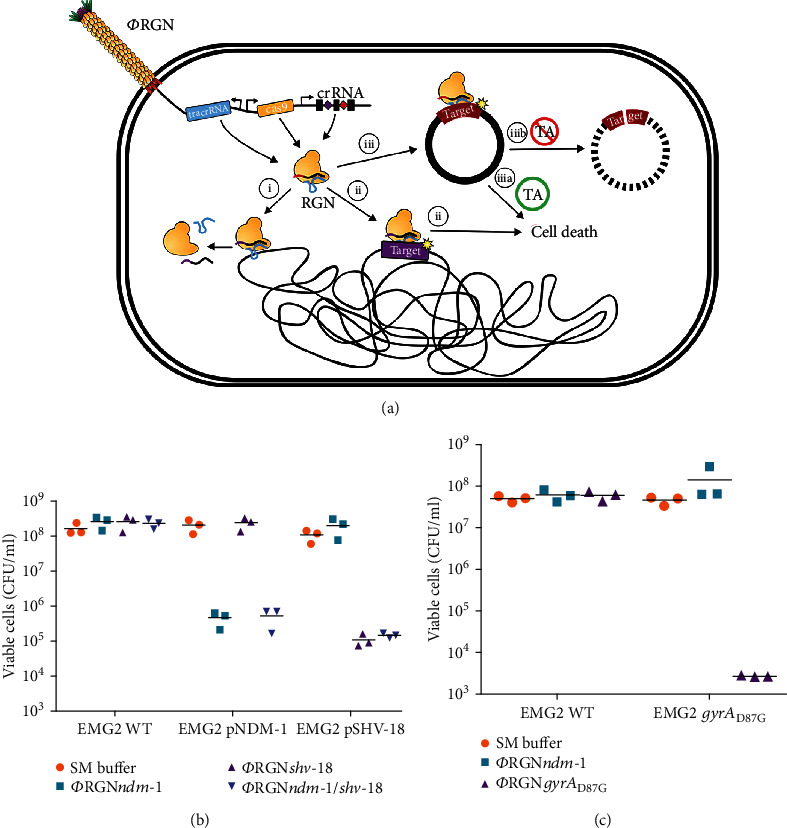
RGN constructs delivered by bacteriophage particles (*Φ*RGN) exhibit efficient and specific antimicrobial effects against strains harboring plasmid or chromosomal target sequences. (a) Bacteriophage-delivered RGN constructs differentially affect host cell physiology in a sequence-dependent manner. (b) Treatment of EMG2 wild-type (WT) or EMG2 containing native resistance plasmids, pNDM-1 (encoding *bla*_NDM-1_), or pSHV-18 (encoding *bla*_SHV-18_), with SM buffer, *Φ*RGN*_ndm-1_*, *Φ*RGN*_shv-18_*, or multiplexed *Φ*RGN*_ndm-1/shv-18_* at a multiplicity of infection (MOI) ~20 showed sequence-dependent cytotoxicity as evidenced by a strain-specific reduction in viable cell counts (*n* = 3). CFU: colony-forming units. (c) *E. coli* EMG2 WT or EMG2 *gyrA*_D87G_ populations were treated with SM buffer, *Φ*RGN*_ndm-1_*, or *Φ*RGN*gyrA*_D87G_ at MOI ~20, and viable cells were determined by plating onto Luria-Bertani agar (*n* = 3) [231]. *Copyright © 2014, Springer Nature.*

Citorik et al. utilized their laboratory-developed phage-delivered CRISPR-Cas9 system (Figure 6) to specifically remove antibiotic-resistant genes such as NDM-1 which allows bacteria to develop resistance to a variety of *β*-lactam antibiotics (Figure 6(a)) [231]. In a group of three different drug-resistance *E. coli* strains, they were able to selectively eliminate the targeted strains while maintaining the integrity of other bacteria (Figures 6(b) and 6(c)). Bikard et al. explored the same system in inhibiting *S. aureus* [232]. Destruction of *S. aureus* plasmids with resistance genes was achieved without damaging the nontoxic *Staphylococci*. The CRISPR-Cas9 system had shown a good effect of killing *S. aureus in vivo* in the mouse skin colonization model.

**Figure 7:**
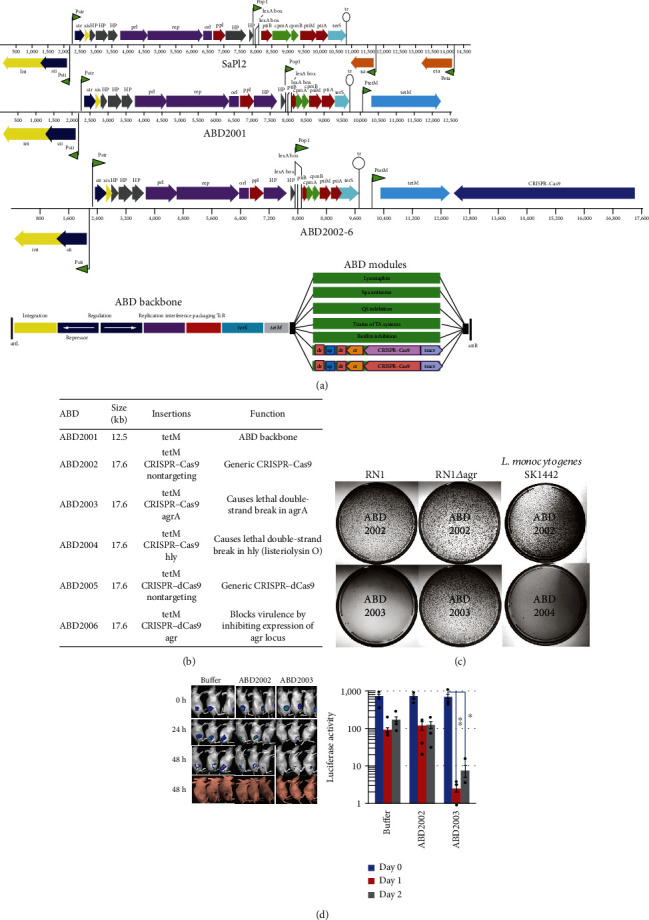
The structures of antibacterial drones (ABD) and their activities *in vitro* and *in vivo*. (a) Genetic maps of SaPI2 and its ABD derivatives. (b) ABD constructs. ABD2001 was derived from the prototypical SaPI2 by deleting toxin genes *tst* and *eta* and the capsid morphogenesis genes *cpmA* and *cpmB*. ABD2002–2006 were derived from ABD2001 by the insertion of the listed genes. (c) Killing of *S. aureus* by ABD2003 and of *L. monocytogenes* by ABD2004. Suitable dilutions of ABD2002, ABD2003, or ABD2004 particle preparations were mixed with RN1, RN1*∆agr*, or *L. monocytogenes* SK1442, plated on Tc5, and incubated at 37°C for 48 h. (d) Blockade of SC murine infections by ABDs [234]. *Copyright © 2018, Springer Nature.*

However, phage-based delivery systems are still inadequate in terms of effectiveness and safety [233]. More recently, researchers have engineered “pathogenicity islands” (PIs) from bacterial DNA, which are the genes unique to the pathogen that evolved from the virus and stays permanently in the virulent bacteria. A CRISPR/Cas9 module capable of specifical cleavage of the *S. aureus agr* gene was then added to cause lethality to the bacteria. The CRISPR–dCas9 module targeted the *agr* P2 and P3 gene to block virulence, creatively transformed PIs into “antibacterial drones” (ABDs) (Figures 7(a) and 7(b)) that prevent *S. aureus* infections [234]. The results showed that these constructed ABDs performed specific killing activities against several *S. aureus* and *Listeria monocytogenes* strains (Figure 7(c)). When mice were injected with a fatal staphylococcus, the genetically modified *S. aureus* PIs killed the pathogenic bacteria and increased the survival rate of infected mice (Figure 7(d)).

At present, two biological companies (Locus Biosciences and Eligo Biosciences) are developing advanced antibacterial therapies with the CRISPR-Cas system. Unlike CRISPR-Cas9, they use CRISPR-Cas3, because the latter can effectively remove the long segment of DNA at a target position in the genome, which is not easy to be achieved by the traditional CRISPR-Cas9 system [235, 236]. Locus Biosciences further exploited the unique properties of CRISPR-Cas3 to target and irreversibly destroy bacterial DNA to kill target bacteria or eliminate specific bacterial populations [237]. Eligo Bioscience focuses on the usefulness of CRISPR-Cas3, hoping that it will not only successfully kill more and more superbugs but also prevent the emergence of superbugs in the future. Though clinical trial has not been done yet, the company has successfully used CRISPR-Cas3 to cure mice infected with two different *E. coli* strains [238].

### 6.2. CRISPR-Responsive Smart Materials

Cas9 is the most in-depth and widely used Cas enzyme thus far and has great application prospects in gene editing and disease treatment [239–241]. However, CRISPR-Cas9 lacks an enzyme-active domain that cleaves single-stranded nucleic acids and cannot be used for the detection of pathogenic infection *in vitro* [242]. In contrast, Cas12a has an additional enzymatic domain, which can be activated to cleave single-stranded substrate ssDNA when recognizing the target gene sequence of a pathogen or tumor. After the domain is being activated, the enzyme will release a fluorescent reporter group that is linked with ssDNA. The latter sequence information can be transformed to a fluorescence signal [243]. This feature has successfully achieved the sensitivity that cannot be achieved by ordinary real-time quantitative PCR and get rid of the dependence on a real-time quantitative PCR instrument as it targets known sequences of pathogens.

**Figure 8:**
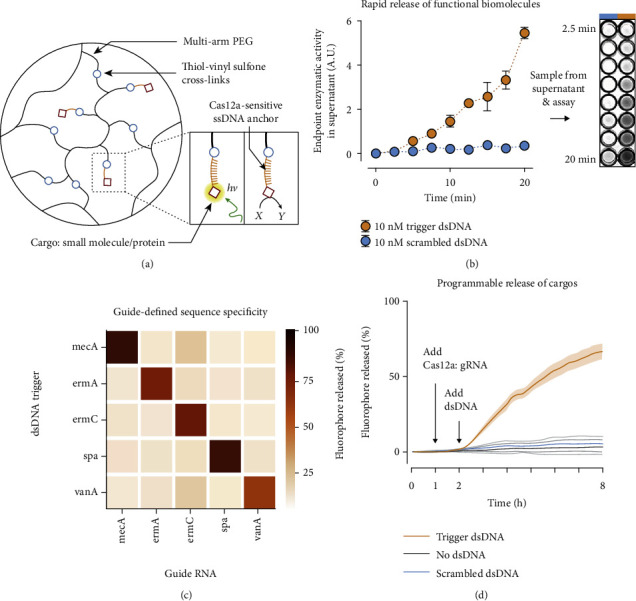
Cas12a-mediated release of small molecules and enzymes from PEG hydrogels. (a) ssDNA acts as a cleavable linker for attaching payloads to an inert PEG matrix. *hν*: light energy. (b) Activation of Cas12a and fluorophore release (*t* = 8 hours) is defined by the complementarity between a dsDNA sequence and the gRNA of Cas12a. (c) Functional enzymes can be anchored into the hydrogel and released by Cas12a in sufficient quantities for visual detection in an HRP activity assay within minutes. A.U.: arbitrary units. (d) Release of a tethered fluorophore by Cas12a is initiated only upon the introduction of a specific dsDNA trigger and not a scrambled dsDNA control sequence [244]. *Copyright © 2019, The American Association for the Advancement of Science.*

English et al. have creatively integrated CRISPR-Cas12a technology into DNA hydrogels (Figure 8) [244]. The Cas12a-gRNA can specifically recognize foreign DNA and activated Cas12a to cleave target dsDNA and proximal indiscriminate ssDNA. Data showed that the DNA hydrogel was gradually disintegrated based on the response to the targeted cleavage of dsDNA and can be used for the controlled release of drugs/antibiotics, nanoparticles, and even cells (Figure 8(a)). The hydrogel structure can respond to any targeted DNA sequence as gRNA within the hydrogel targets genes involved in the antibiotic-resistance mechanisms of *S. aureu*s, such as *ermA*, *ermC*, *spa*, and *vanA* (Figure 8(b)). The hydrogel system required only nanomolar or even picomolar concentration of targeted DNA to achieve efficient cutting of the CRISPR-Cas12a system (Figure 8(c)) [244]. The authors also reported on the controlled release of small molecule drugs or proteins in multiarm polyethylene glycol hydrogels, gold NPs, and even live-cell controlled release polyacrylamide hydrogels, which subtly convert biological information into macroscopic changes in material properties (Figure 8(d)). This platform shows a potential application value in medical analysis and environmental monitoring and has a promising future in the application of POT.

In summary, though the treatment of antimicrobial agents based on CRISPR-Cas systems still has a long way to go from laboratory to clinical applications, this technical strategy is novel and has great potential for combatting AMR. Once the technique is established, it will change the way we treat MDR infections in patients. Besides that, it also represents a novel and powerful way in catalyzing the change of human microbial composition and helps to develop new treatments for key diseases of drug-resistant bacterial infections.

## 7. Microbiota Therapy in Combatting AMR

The role of microbiota in regulating human health and disease status has received increasing attention in recent years. The destruction of intestinal flora has been proven to be involved in the pathogenesis of many infectious diseases [245, 246]. Manipulating and engineering human microbiota for combatting AMR are an attractive option for POT.

Recent studies have found that drug resistance is transmitted via gut bacteria even without the use of antibiotics. Persistent bacteria, also known as persisters, are the main culprits for the spread of AMR [247]. Bacteria like *Salmonella* carry resistance genes, allowing them to survive in antibiotic treatment and remain undetected for months. They are in a temporary dormant state and can minimize their metabolism, preventing the antibiotics from killing them. If the condition favors bacterial survival, dormant *Salmonella* can transfer their resistance gene to other bacteria of the same species or even to other species such as *E. coli* in the gut and infection can then reemerge [247]. Fecal microbiota transplantation (FMT) therapy can circumvent the risk of drug resistance caused by antibiotic treatment, which is mainly to rebuild the intestinal tract of the patient by transplanting the intestinal flora from human feces into the intestine of the patient. This therapy can also be used for the treatment of diarrhea, intestinal microflora disorder, and other diseases [248–250]. The implication of fecal transplantation is to reestablish a normal intestinal microecosystem. Utilizing the FMT for the treatment of *C. difficile* colitis is the most in-depth application case for clinical research [251–253]. One of the causes of *C. difficile* colitis is the inappropriate use of broad-spectrum antibiotics, causing damage to the normal intestinal microbiota [254, 255]. A clinical study demonstrated that the initial cure rate reached 91.2%, and the recurrence rate was only 5.5% after 611 patients were treated with FMT, showing that it is a fairly good treatment [256]. It is important to first screen suitable fecal donors before the FMT can be initiated. Fresh feces of healthy donors are obtained and transplanted into patients' intestines and stomach through patients' mouth, nose, or anus [257, 258]. Most treatment operations have been performed through enemas at present. The applicability of FMT remains a concern as there are deficiencies in the effectiveness and safety of the methodology. Recently, the US Food and Drug Administration (FDA) reported that a patient died as a result of FMT treatment and warns that there is a serious risk for receiving FMT treatment, which is the spreading of bacteria that are resistant to various drugs [259]. More research is required to address the issues related to FMT and validate the efficacy of the treatment before being expanded to the public.

**Figure 9:**
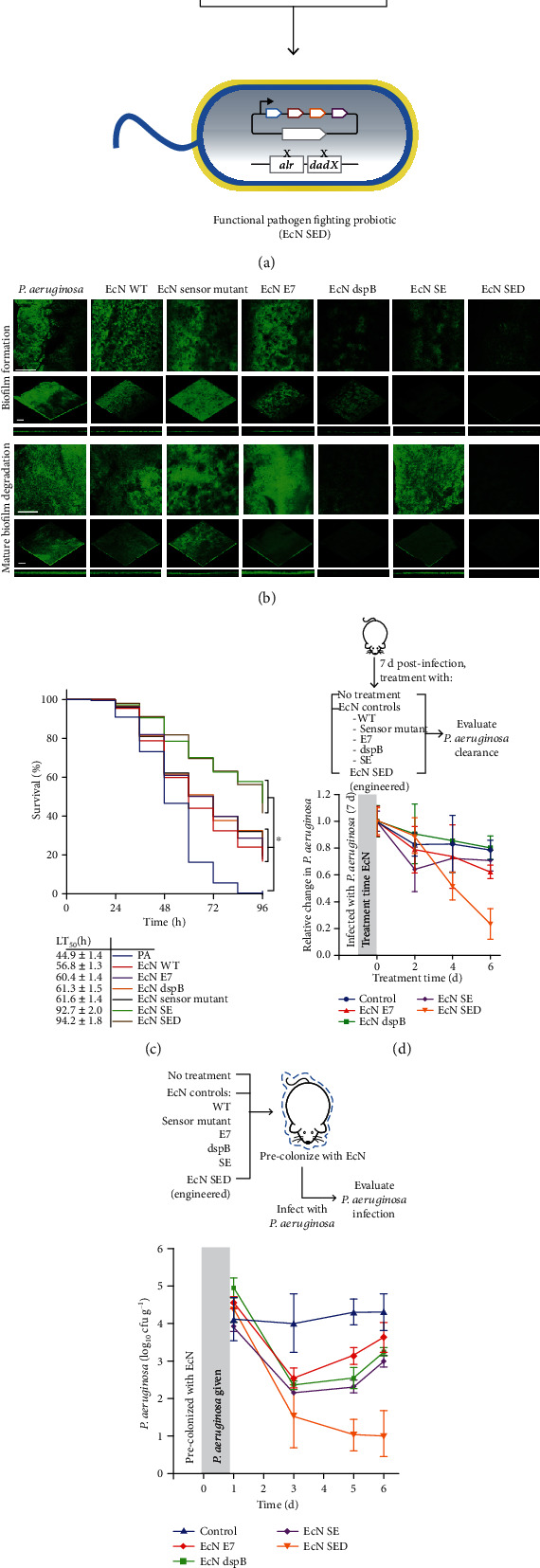
Schematic diagram of constructing a functional probiotic strain and its activity against *P. aeruginosa* infection. (a) Vector-host system created in the *E. coli* Nissle strain by auxotrophic complementation to stabilize plasmid retention. (b) *P. aeruginosa* was cocultured with engineered EcN for 20 h to evaluate its effect on biofilm formation. (c) The survival rate of each treatment group was quantified until all the nematodes in the infection group had died (96 h). (d) Evaluation of the engineered probiotic strain in a mouse infection model. (e) Prophylactic activity of engineered EcN against *P. aeruginosa* infection [264]. *Copyright © 2017, Springer Nature.*

The use of probiotics to treat various infectious diseases is another alternative of POT. Probiotics play important roles in the fight against pathogens in humans. They mainly inhibit or exclude the growth of other harmful microorganisms by competing for nutrients or adhesion space, releasing antibacterial compounds, stimulating the host immune system, and enhancing the intestinal barrier function [260, 261]. In a study of the effects of probiotics on the incidence of *C. difficile*-associated diarrhea for children and adults in hospital and outpatient settings, the use of *Lactobacillus*, *Saccharomyces*, and a mixture of probiotics significantly reduced the incidence 63.7%, 58.5%, and 58.2%, respectively [262]. A modified strain of *E. coli* Nissle 1917 has been found to release toxins that selectively eliminated *P. aeruginosa* [263]. Researchers further engineered this probiotic strain to confer a gene that can disrupt the stability of the *P. aeruginosa* biofilm (Figures 9(a) and 9(b)). The engineered probiotic strain showed an impressively prophylactic and therapeutic activity against *P. aeruginosa* in two gut-infected models, mice, and *C. elegans* (Figures 9(c)–9(e)). Engineered probiotics represent a more primitive way to combat against AMR and have shown great potential in preventive and therapeutic activity against intestinal infections [264]. Nevertheless, more investigation or clinical studies are needed to further evaluate and understand the mechanisms involved in fighting against AMR.

In summary, microbiota therapy is an attractive option for the prevention and resolution of AMR. On the one hand, the treatment is not easy to cause drug resistance; on the other hand, it will not destroy the human microbiota or increase the possibility of reinfection. However, the lack of systematic understanding of the complex genome and phylogenetic diversity of human microbiota is a key challenge for this therapy currently. Therefore, it is necessary to have a deeper understanding of the complex role of microbiota in the pathogenesis of specific diseases. In addition, although microbiota therapy has been in the late-stage of clinical trials to prevent CDI recurrence, the manufacturing process of live bacteria products is complex and expensive, and there is still a lack of proven theoretical basis or models to support the search for appropriate doses. All these make microbiota therapy still need to move forward cautiously in the fight against AMR.

## 8. Concluding Remarks and Future Perspectives

The great success of conventional antibiotics has greatly improved people's quality of life, but drug resistance poses serious threats to the public health nowadays. Conventional antibacterial treatment faces many limitations, e.g., the treatment regimens available for MDR pathogens are depleted and the available antibiotic-specific activity is lacking. As a result, alternatives to traditional antibiotics are in urgent need, i.e., POT strategies targeting either specific bacterial species or strains or host infection sites, to address the growing clinical embarrassments of available antibiotics. Conjugation of existing antibiotics not only provides conventional antibiotics with dual-targeting and synergistic antibacterial activities to improve their pharmacokinetic parameters but also reduces their sensitivity to degrading enzymes and efflux systems. Despite these advantages, the efficacy of conjugated antibiotics is expected to prove superior to or at least equal to that of combination therapies, and the mechanisms of these conjugates against drug resistance still need to be systematically studied. AMPs are less likely to induce drug resistance compared with conventional antibiotics, but more efforts are required to be done in this research field, especially clinical trials, to evaluate the efficacy and safety of AMPs in fighting against bacteria. mAbs have been approved for the treatment and prevention of some common bacterial infections, but their widespread applications are constrained by production costs, expiration date, and individual differences among patients. NPs have unique advantages in the targeted delivery of antibiotics. Current researches focus on the basic strategy of targeted delivery of NPs. The clinical application of antimicrobial NPs is rare at present, and we foresee that it has a bright future in the treatment of infectious diseases. The CRISPR system has made significant progress in the fight against drug-resistant diseases, but there is still room for improvement for safer and more efficient drug delivery systems. Recent research has also developed new strategies for targeting delivery systems, i.e., toxin-intein antimicrobial that can specifically eliminate pathogenic bacteria without inducing damage on the host's beneficial microbiome [265]. This is expected to be a new strategy and trend for treating bacteria-related disorders and AMR. A deep understanding of microbial dynamics and metabolic interactions is important for the development of inhibiting or conquering opportunistic pathogens.

Each new therapy has its own advantages and limitations. For example, AACs are chemically synthesized, and their transportation and storage do not require special equipment, so they have advantages over the price of other treatments. But in some ways, this treatment strategy can only slow down the development of drug resistance. For some infected patients who need precision or personalized care, therapies based on antibody or CRISPR system may be the next frontier directions because they have a highly precise targeting effect. However, these two therapies are relatively expensive, antibody-based therapy should avoid antibody enhancement effects, and CRISPR system-based therapy should pay attention to off-target effects. AMPs are generally positively charged cationic peptides with broad-spectrum antibacterial activity. Their molecular weights are between traditional antibiotics and antibodies, and the cost of synthesis is relatively high. Minimizing the degradation and toxic effects on mammalian cells in order to obtain a large enough treatment window remains the main challenge for the use of AMPs. The major obstacles to the clinical application of antimicrobial NPs are their safety and cytotoxicity concerns, such as metabolism, clearance, and mode of action, which must be further evaluated, since the interaction of NPs with cells and tissues is still poorly understood. Another obstacle to overcome is the development of affordable mass manufacturing methods for NPs. In spite of this, they have a bright future in achieving drug delivery at specific infection sites in reducing off-target effects, reducing unnecessary toxicity, and improving the therapeutic efficacy of drugs. Microbiota therapy is an indirect treatment strategy which does not inhibit or kill bacteria but play a role by regulating or interacting with complex microorganisms. Thus, traditional antimicrobial measurements such as MIC assay cannot be used to measure its therapeutic effect. Therefore, finding the evaluation method of the appropriate dose still needs to be explored. In addition, unlike those therapies mentioned above, tailor-made treatments are currently difficult to achieve and may require a comprehensive understanding of the underlying mechanisms and patient factors of the microbiota. Undeniably, these POT strategies cannot completely replace traditional antibiotic therapy but they can act as coadjuvants to fight against AMR. Therefore, therapies based on antibiotics and their combination with AMPs, antibodies, nanotechnology, and CRISPR systems are worth exploring to discover their full potential. Most of these treatment pathways are still under development and requires time, resources, and efforts for the further advancement.

## Figures and Tables

**Table 1:** Examples of AACs.

Antibiotic conjugates		Evaluated condition	Proposed mechanism of action	Reference
Quinolone/fluoroquinolone	Quinolone-oxazolidinone (MCB3681)	Gram-positive bacterial infections	Inhibit the initiation of bacterial protein biosynthesis	[26, 27]
	Quinolizine-rifamycin (cadazolid)	*C. difficile* infections	Inhibit protein and RNA synthesis	[28–30]
	Fluoroquinolone-oxazolidinone (CBR-2092)	Gram-positive bacterial infections	Inhibit the bacterial DNA replication and DNA-dependent RNA synthesis	[31, 32]

Aminoglycoside	Neomycin B-ciprofloxacin	Gram-negative bacteria and Gram-positive MRSA infections	Inhibit the activity of DNA gyrase, topoisomerase IV, and protein synthesis	[33]
	Tobramycin-moxifloxacin	*Pseudomonas aeruginosa* infections	Enhance the permeability of antibiotics to the outer membrane of pathogenic bacteria	[34, 35]
	Neomycin-sisomicin	Aminoglycoside-resistant bacteria infections	Inhibit protein synthesis by binding to 16S rRNA	[36, 37]

*β*-Lactamase inhibitor	Ceftazidime-avibactam	Complicated urinary tract infections	Interfere with bacterial cell wall and peptidoglycan synthesis	[22, 38]
	Meropenem-vaborbactam	Complicated urinary tract infections and acute pyelonephritis	Vaborbactam potentiates the activity of meropenem, inhibiting the cell wall synthesis and peptidoglycan synthesis	[20, 39, 40]
	Imipenem-relebactam	Gram-negative bacterial infections	Relebactam prevents the hydrolysis of imipenem, exerting imipenem's bactericidal effect	[41, 42]

Macrolide	Azithromycin-sulfonamide	Macrolide-resistant *Streptococcus pyogenes* and *Streptococcus pneumoniae* strains	Inhibit mRNA translation and bacterial metabolic processes	[43, 44]

**Table 2:** Some selected AMPs in the latest stage of clinical research.

Peptide	Description	Evaluated condition	Clinical trial phase	Company	Proposed mechanism of action
AA139	Originates from arenicin-3	Urinary tract infection	Phase I	Adenium Biotech	Interruption of phospholipid transportation pathways, membrane dysregulation
AB103	A peptide mimetic of CD28	Necrotizing soft tissue infections	Phase III	Atox Bio	Attenuate cd28 signaling during bacterial infection
Dalbavancin	Semisynthetic lipoglycopeptide	Gram-positive osteoarticular infections	Phase IV	Infectious Diseases Physicians, Inc.	Inhibit transglycosylation and transpeptidation for cell-wall synthesis
Pexiganan	Analogue of magainin-2	Infected diabetic ulcers	Phase III (failed)	Dipexium Pharmaceuticals, Inc.	Cell membrane disruption
LL37	A 37 amino acid cationic peptide of the hCAP18 protein	Venous leg ulcers and diabetic foot ulcers	Phase IIa	Promore Pharma	Modulate the inflammatory phase
SAAP-148	An LL-37-derived peptide	Atopic dermatitis and methicillin-resistant staphylococcus aureus infections	Preclinical	Madam Therapeutics	Cell membrane modulators
Oritavancin	A semisynthetic lipoglycopeptide	Acute bacterial skin and skin structure infections	Phase III	Melinta Therapeutics, Inc.	Inhibit cell wall biosynthesis
PAC-113	A 12-amino acid peptide derived from the histatin	Candidiasis infection	Phase IIb	Pacgen Biopharmaceuticals Corporation	Cell membrane permeability enhancers; reactive oxygen species stimulants
SGX942	Synthetic 5-amino acid peptide	Oral mucositis	Phase III	Soligenix	Target the intracellular control pathways
Murepavadin (POL7080)	A 14-amino-acid cyclic peptide based on protegrin-1	Pseudomonas infections	Preclinical	Polyphor Ltd.	Target the Gram-negative bacterial outer membrane proteins
Novexatin (NP213)	A cyclic arginine-based heptamer	Toenail infections	Phase I/IIa	NovaBiotics Ltd.	Disrupting bacterial cell membranes
hLF1-11	The first eleven amino acids of the natural human lactoferrin	Infection of bone marrow stem cell transplantations	Phase II	Am-Pharma	Modulation of the immune system
Brilacidin	A mimic of defensin	Acute bacterial skin and skin structure infection	Phase III	Innovation Pharmaceuticals Inc.	Disrupting bacterial cell membranes
PXL01	A synthetic peptide derived from the human lactoferricin peptide	Postsurgical adhesions and scars	Phase III	Promore Pharma	Immunomodulation and enhancement of fibrinolytic activity
OG716	A derivative of mutacin 1140	*Clostridium difficile* infection in enteritis	Preclinical	Oragenics, Inc.	Transmembrane lipid II-mediated pore formation
Omiganan	A synthetic 12 amino acid peptide, analogue of indolicidin	Catheter infections	Phase II	Cutanea life sciences	Cell membrane permeability enhancers

**Table 3:** Some antibodies against pathogenic bacteria in clinical research.

Antibody	Evaluated condition	Target	Clinical trial phase	Method of generation	Reference
MEDI4893	*S. aureus* pneumonia	Alpha toxin	Phase IIb	Hybridoma technology	[178–180]
ASN100	*Staphylococcal infections*	Alpha toxin, leukocidins	Phase II	Yeast surface display	[181, 182]
AR-301	*S. aureus* lung infections	Human leukocyte antigen	Phase III	Screening human B-cells of convalescent pneumonia patients	[183]
514G3	*S. Aureus bacteremia*	SpA	Phase II	B-cell isolation	[184, 185]
AR-101	*P. aeruginosa* infections	LPS	Phase IIa	Screening of the B-cell repertoire	[186]
MEDI3902	*P. aeruginosa* pneumonia	PcrV and Psl	Phase II	Phage display	[187–189]
KB001-A	*P. aeruginosa* infections	PcrV	Phase II	Hybridoma technology	[190, 191]
Raxibacumab	Anthrax	Protective antigen	FDA approved	Phage display	[192]
Anthim	Anthrax	Protective antigen	Phase I	Mouse hybridoma	[193]
Bezlotuxumab	*C. difficile* infections	Toxin B	FDA approved	Mouse immunization	[194, 195]
Shigamabs	Shiga toxin-producing infections	Shiga toxin 1 and 2	Phase II	Mouse hybridoma	[196]
